# Experiences of living with chronic myeloid leukaemia and adhering to tyrosine kinase inhibitors: A thematic synthesis of qualitative studies

**DOI:** 10.1016/j.ejon.2020.101730

**Published:** 2020-04

**Authors:** Ann Hewison, Karl Atkin, Dorothy McCaughan, Eve Roman, Alex Smith, Graeme Smith, Debra Howell

**Keywords:** Adherence, Chronic cancer, Chronic myeloid leukaemia, Treatment, Qualitative synthesis, Tyrosine kinase inhibitors, Survivorship

## Abstract

**Purpose:**

To investigate the experiences of adults living with chronic myeloid leukaemia and treated with tyrosine kinase inhibitors, with particular reference to factors influencing adherence.

**Methods:**

A thematic synthesis of all published qualitative studies examining adults with chronic myeloid leukaemia, receiving tyrosine kinase inhibitors. Eligible publications were identified by searching five electronic databases using defined criteria. The synthesis involved complete coding of extracted data and inductive theme development.

**Results:**

Nine studies were included and three overarching themes defined. Overarching themes were: 1) Disease impacts whole life; 2) Disease management strategies; and 3) Valued aspects of care. Side-effects often required physical and psychological adaptation. Patients developed individual decision-making processes to promote adherence and manage side effects. Unintentional non-adherence occurred due to forgetfulness and system failures. Intentional omission also occurred, which together with side effects, was unlikely to be reported to healthcare professionals (HCPs). HCP reassurance about missed doses could reinforce non-adherence. Information needs varied over time and between individuals. Knowledge among patients about treatment was often lacking and could lead to misunderstandings. Patients valued psychological support from HCPs and suggested an individualised approach, facilitating discussion of symptoms, adherence and their perspectives about living with chronic myeloid leukaemia, would improve care.

**Conclusions:**

Patients with chronic myeloid leukaemia experienced significant side-effects from treatment and changes to their psychological and physical well-being. They developed their own strategies to manage their disease and treatment. This should be recognised in interventions to improve education, support and the delivery of care that is compassionate and adequately resourced.

## Highlights

• The first qualitative synthesis of chronic myeloid leukaemia patient experiences.
• Contrary to trial evidence, CML has significant physical and psychological impact.
• Patients develop strategies to manage treatment, often without professional input.
• Patients value psychological support, professionals require resources to give this.

## 1 Introduction

Chronic myeloid leukaemia (CML) is a haematological malignancy arising when bone marrow stem cells produce excessive and abnormal white cells. Most people with CML have the Philadelphia chromosome which carries the defective *BCR-ABL₁* gene, enabling production of a tyrosine kinase enzyme which stimulates the disease process (Frazer et al., 2007). It is characterised by a chronic, accelerated and blast phase, with most diagnoses made in the chronic phase and commonly associated with anaemia and splenomegaly (Jabbour and Kantarjian, 2018). A rare disease (European incidence 1–2/100,000 population), with an average age at diagnosis of around 57 years, CML is more common in men than women (Brunner et al., 2013; Pulte et al., 2013; Rohrbacher and Hasford, 2009; Smith et al., 2011; Visser et al., 2012). Incidence of CML does not differ by ethnic origin, geographical region or socioeconomic status (Hehlmann et al., 2007; Smith et al., 2011).

The introduction of oral tyrosine kinase inhibitors (TKIs: targeted therapies given orally to block cancer cell growth) at the turn of the current century transformed CML from a rapidly fatal disease, to an illness with a chronic trajectory. Imatinib (or Gleevec/Glivec) was the first TKI to be introduced, followed by a range of ‘second generation’ drugs. Survival has since improved to the extent that European rates are now similar to those of the general population (Björkholm et al., 2011; Smith et al., 2014). Response to TKIs is described as “the most important prognostic factor” for CML management in the European LeukaemiaNet recommendations (Baccarani et al., 2013) and has the greatest effect on survival. Importantly, several studies examining treatment have identified a link between adherence and response (Almeida et al., 2013; Ganesan et al., 2011; Marin et al., 2010; Noens et al., 2009), with influencing factors including: drug dose, time since diagnosis, treatment duration, comorbidity, clinician/patient relationships and patient understanding of CML (Gater et al., 2012; Noens et al., 2014). Since more people are living with the long-term effects of CML (Atallah and Ritchie, 2018), health related quality of life (HRQOL) and symptom burden have gained particular importance. Unfortunately, however, significantly worse outcomes are reported in people with CML compared to the general population (Efficace et al., 2011; Phillips et al., 2013); a situation which can affect adherence (Marin et al., 2010).

Research examining these issues has been criticised for taking a “reductionist biomedical” approach, measuring only objective predictors of non-adherence (i.e. disease and treatment related factors), rather than investigating the role of patients’ beliefs, experiences and social situation (Gater et al., 2012). As Sabaté (2003) highlight in their key World Health Organisation (WHO) report, viewing the patient as having individual responsibility for adherence ignores contextual factors which impact upon it, such as socioeconomic and health system issues. More recently, however, qualitative studies have examined broader patient experiences (e.g. Graffigna et al., 2017; Lim et al., 2017). The pragmatic aims of the current study are to: 1) explore how individuals perceive and describe their experiences of taking long-term TKIs, with particular reference to adherence, side effects and quality of life; and 2) generate evidence that can be used to guide clinical practice.

## 2 Methods

Although the first part of the synthesis is an open question (to explore the CML experience), suggesting iterative or interpretive approaches were appropriate (Barnett-Page and Thomas, 2009; Dixon-Woods et al., 2006, 2005; Paterson, 2012), the second part (to inform clinical practice) is more pragmatic. Various methods of qualitative synthesis were investigated to find a methodological approach that could incorporate both aspects of the research question, with thematic synthesis considered the most appropriate. Thematic synthesis is a realist approach, which permits an open research question and also reflects our pragmatic aim. In this way, it is comparable to the idea of “subtle realism” (Hammersley, 1992), which accepts that there is a shared reality outside of us, but that one can only know this reality through the minds and perspectives of individuals. Other factors, such as researcher experience and background, available resources and type of data also suited the thematic synthesis approach. Methods were guided by key references (Braun and Clarke, 2013; Thomas and Harden, 2008), as recommended (Barnett-Page and Thomas, 2009; Booth et al., 2016; Flemming, 2007; Paterson, 2012), and are presented below in accordance with the ENhancing Transparency in REporting the synthesis of Qualitative research (ENTREQ) statement (Tong et al., 2012).

### 2.1 Search strategy, eligibility and screening

A systematic search of: “chronic myeloid leukaemia *or* chronic myeloid leukaemia *or* leukaemia myelogenous chronic BCR-ABL positive” *and* “patient satisfaction *or* patient experience *or* qualitative research” was conducted within MEDLINE, CINAHL, PsycINFO, Social Sciences Citation Index: Web of Science, and Google Scholar. Electronic alerts were set up in each site, with Scopus used to check citations. The initial search was conducted in 2016, with papers screened for eligibility (see Table 1 for criteria) using the study abstract or full text.

**Table 1:** Eligibility criteria.

	Inclusion	Exclusion
**Participant characteristics**	CML diagnosis Aged ≥18 years Males and females Chronic phase Long-term TKI use (i.e. lifelong) Outpatient management Any geographical location	Aged ≤18 years Accelerated/blast phase Receiving end-of-life care Not treated with TKIs Inpatient management
**Type of study**	Qualitative	Clinical trials/quantitative Systematic reviews Non-English language

**Fig. 1:**
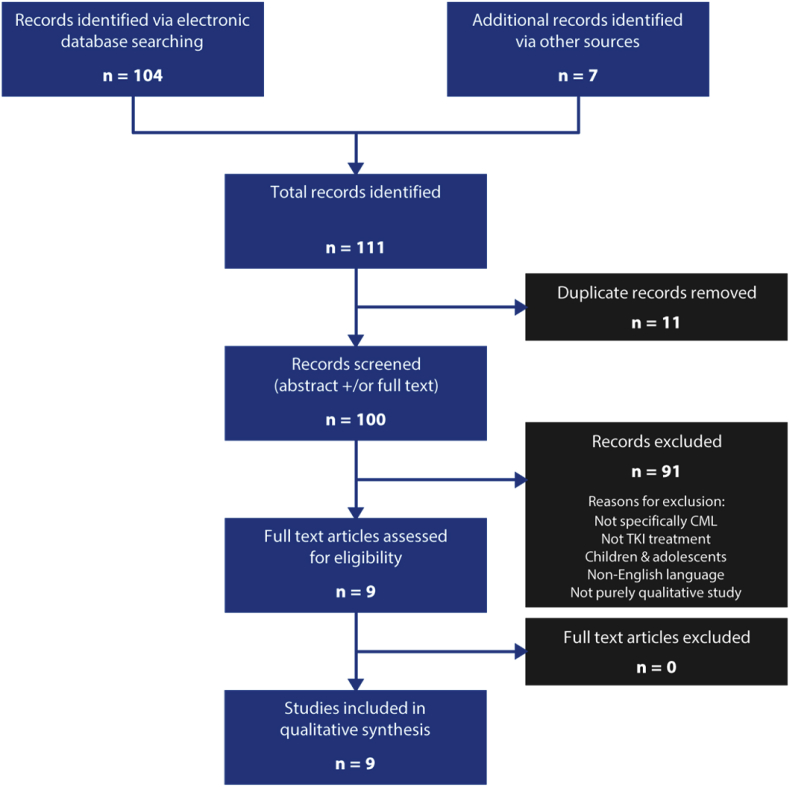
Screening process and identification of eligible studies.

Initial data base searching and citation searches led to the identification of 104 studies, with 7 additional papers found via database alerts (up until September 2019). After the removal of duplicates, 100 studies were screened and 91 removed. Nine studies emerged as eligible, as shown in the PRISMA flow chart in Fig. 1 (Liberati et al., 2009). Table 2 provides summaries of the included studies. Strengths and limitations of eligible studies were appraised by two researchers (AH, DM) using a quality assessment tool (Hawker et al., 2002). Each study was examined using this tool (Hawker et al., 2002) to allocate gradings (‘poor’, ‘fair’ and ‘good’), as shown in Table 3. Strengths were noted in the reporting of findings, which ranged from descriptive to conceptual accounts, with quotations being consistent and illustrative of results and themes. Weaknesses were noted in most studies: several did not describe the relationship between researchers and participants or inclusion/exclusion and sampling criteria; others used a theoretical framework but did not report how this was applied during data analysis.

**Table 2:** Summary of included studies.

Author/year	Population/country	Participants (N, age, sex)	Research question	Data collection	Research approach/analysis
Eliasson et al. (2011)	CML patients attending hospital, UK	N = 21 Age 33-70 Male 11, Female 10	To explore the experience of CML patients of taking (or not) imatinib as prescribed	In-depth unstructured interviews	Constant comparison
Guilhot et al. (2013)	CML patients from clinical centres and online communities, Brazil, France, Germany, Russia and Spain	N = 50 Age 21-80 Sex not reported	To assess the effects of diagnosis and treatment on patients with CML and offer recommendations for HCPs to better support patients	In-depth, semi-structured interviews with patients and relatives. Diary, photo journal and debriefing interview (Brazil and France only)	Ethnography
Chen et al. (2014)	CML patients attending an oncology outpatient clinic, Southern Taiwan	N = 42 Age 20-80 Male 23, Female 19	To explore CML patients' experiences of treatment with imatinib, and understand perceptions, attitudes and concerns that may influence adherence	Semi-structured interviews	Constant comparison; theme saturation
Wu et al. (2015)	CML patients and HCPs at a specialist cancer centre, Australia	Patients: N = 16 Age 26-71 Male 9, Female 7 Practitioners: N = 10 (4 Haematologists, 3 nurses, 3 pharmacists)	To explore and compare patient experiences with HCP perceptions of imatinib	Semi-structured interviews	Interpretative phenomenological analysis
Bolarinwa et al (2018)	CML patients attending the only hospital providing free imatinib, Nigeria	N = 20 Age 25–56 Male 10, Female 10	To evaluate delayed diagnosis, health-seeking behaviour, medication use and other challenges faced by people living with CML on imatinib	In-depth semi-structured interviews	Grounded theory (until saturation); content analysis of themes
Graffigna et al. (2017)	CML patients in 22 onco-haematological centres, Italy	N = 158 Characteristics not reported	To reconstruct the subjective meaning- process related to CML and explore the psychological impact of suspending therapy	Narrative diaries	Narrative inquiry. Lexicography software analysis and a “purely qualitative analysis” of narratives by hand.
Lim et al. (2017)	CML patients at a tertiary care centre, Northern Malaysia	N = 13 Age 47.8 (mean) Male 8, Female 5	To explore patients' understanding and challenges in taking imatinib and nilotinib	Semi-structured interviews Questionnaire	Content analysis
Boons et al. (2018)	CML patients from a Dutch advocacy group, treated at 9 hospitals, Holland	N = 13 Age 27-73 Male 5, Female 8	To understand reasons for non-adherence and patient need for information and communication	Semi-structured interviews Questionnaire	Mixed methods Qualitative thematic framework analysis

**Table 3:** Quality appraisal of included articles using Hawker et al. (2002).

Author/year	Abstract/title	Introduction/aims	Methods/data	Sampling	Data analysis	Ethics/bias	Findings	Transferability/generalisability	Implications/usefulness
Eliasson et al. (2011)	Good	Good	Fair	Fair	Fair	Fair	Good	Fair	Fair
Guilhot et al. (2013)	Good	Fair	Good	Fair	Fair	Fair	Good	Fair	Fair
Chen et al. (2014)	Good	Fair	Fair	Fair	Poor	Fair	Fair	Fair	Fair
Wu et al. (2015)	Fair	Good	Fair	Fair	Fair	Fair	Fair	Fair	Good
Bolarinwa et al. (2018)	Fair	Fair	Fair	Fair	Poor	Fair	Good	Fair	Fair
Graffigna et al. (2017)	Fair	Fair	Fair	Poor	Fair	Poor	Fair	Poor	Fair
Lim et al. (2017)	Good	Good	Good	Fair	Poor	Fair	Fair	Good	Good
Tan et al. (2017)	Good	Good	Good	Fair	Fair	Fair	Good	Fair	Good
Boons et al. (2018)	Fair	Fair	Fair	Fair	Poor	Fair	Good	Fair	Good

### 2.2 Data extraction and coding

Extracted data included participant quotations, researcher summaries, and analytical concepts and interpretations, which ensured findings were captured clearly (Thomas and Harden, 2008). Thematic synthesis involved complete coding of extracted data, with codes derived inductively, based on the study aims (Braun and Clarke, 2013; Thomas and Harden, 2008). This was carried out manually (AH), with text highlighted and annotated prior to the generation of codes/sub-codes, named to encapsulate “meaning and content” (Thomas and Harden, 2008). Codes were compared across eligible publications, with new entities created and existing fields merged until a coding frame was finalized (Braun and Clarke, 2013). Publications and coding schemes were uploaded into NVIVO, which was used as a retrieval tool for theme development. Themes were developed inductively (AH), based on similarities and differences between codes, with figurative meaning sought via visual mapping and iterative checking, independently assessed by a second researcher (DM). Themes and sub-themes are reported in the Results, represented by patient quotations and excerpts from author-interpretations.

## 3 Results

Characteristics of the nine included studies are shown in Table 2. All were published 2011–2018 and included people receiving imatinib or second line TKIs for CML. Not all studies reported the type of TKI as follows: i) Imatinib: 4 studies; ii) TKI (type not reported): 3 studies; iii) “first and second line TKIs”: 1 study; iv) Imatinib or nilotinib: 1 study. Often the emphasis was on adherence, but studies also explored patient perceptions of CML, disease stage, disease impact and health-seeking behaviour. All publications contained patient interviews and one also included health care practitioners (HCPs) (Wu et al., 2015). Only data from the patient sample in the latter study was used in the synthesis, to comply with eligibility criteria. Studies were located in Europe, Africa, Australia and South East Asia; and used various qualitative methods, including ethnography, interpretative phenomenological analysis and grounded theory.

**Fig. 2:**
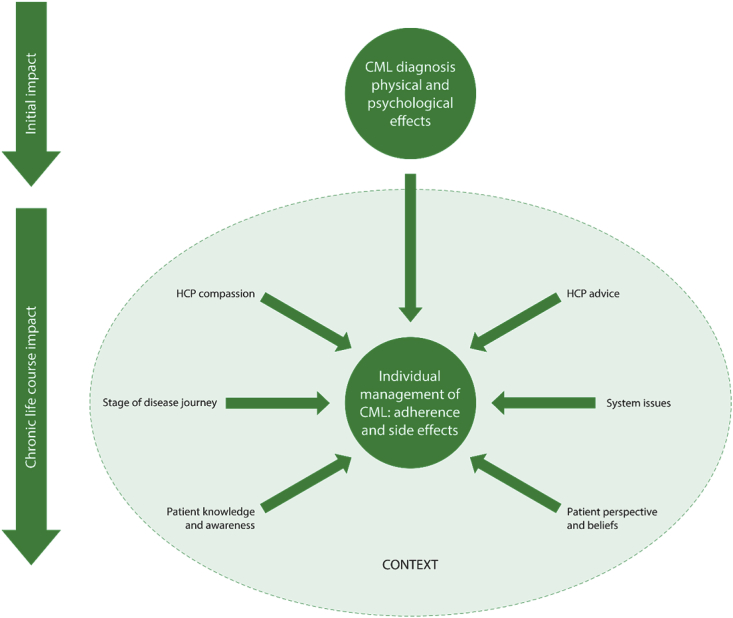
The patient experience of CML in context and over time.

Thirty-eight codes were generated from included studies with three overarching themes: 1) Disease impacts whole life; 2) Disease management strategies; and 3) Valued aspects of care; each of which had multiple sub-themes. Themes and sub-themes are reported in the following section, with verbatim patient quotations and excerpts from author-interpretations, which are clearly marked as such. Themes are also summarised in Fig. 2, which demonstrates how the initial impact of a CML diagnosis influences the way individuals manage their disease and treatment at this time, and the effect of factors arising over the life-course, including hospital care, disease awareness and changing perspectives and beliefs. Overall, Fig. 2 illustrates the individual, situated within the context of what is essentially chronic cancer.

## 4 Theme 1: disease impacts on whole life

This theme relates to the physical, psychological and practical impacts of living with CML, including the effect of this cancer on different areas of life.

### 4.1 Side effects

Side effects from TKI treatment were common and reported as physical or psychological. Physical symptoms commonly included nausea and/or vomiting, pain, skin problems and fatigue. Medication and disease effects were reported as impacting on daily life, usual activities and adherence (Bolarinwa et al., 2018; Boons et al., 2018; Chen et al., 2014; Eliasson et al., 2011; Lim et al., 2017; Tan et al., 2017; Wu et al., 2015):

*“Tiredness of colossal, you know—I've got a young family and just sort of trying to keep up with the daily routine of that is not easy.*” (Wu et al., 2015, p258)

*“… I don't want to take it, because it makes me feel sick. And the next day I'd feel a bit better, because I'd not had them* ……*I consciously didn't take it. Because I didn't want to take it …*” (Eliasson et al., 2011, p629)

Psychological effects included low mood, but also heightened general health awareness and changes in self-identity through a lessening of self-efficacy and the change from individual to patient (Chen et al., 2014; Graffigna et al., 2017; Guilhot et al., 2013):

*“I was a young man at that stage, I was full of energy and enthusiasm. Full of projects for the future. I felt that I was unbeatable. The diagnosis initially destroyed me and my perceived strength*” (Graffigna et al., 2017, p2748, )

Side effects could, however, be mild, or managed by switching to second generation TKIs (Bolarinwa et al., 2018; Chen et al., 2014; Eliasson et al., 2011; Guilhot et al., 2013; Lim et al., 2017).

### 4.2 Adapting daily life

Many areas of life were affected by CML and its treatment; including employment, leisure activities and family roles (Bolarinwa et al., 2018; Boons et al., 2018; Chen et al., 2014; Eliasson et al., 2011; Graffigna et al., 2017; Guilhot et al., 2013; Lim et al., 2017; Wu et al., 2015). Practical concerns about employment and financial matters were reported by several patients, in relation to side effects of TKIs, the need for frequent hospital appointments or stigma relating to the disease (Chen et al., 2014; Graffigna et al., 2017; Guilhot et al., 2013; Tan et al., 2017) In response, patients adapted their routines to cope and manage, including changing work commitments and/or stopping hobbies (Boons et al., 2018; Chen et al., 2014; Eliasson et al., 2011; Graffigna et al., 2017; Guilhot et al., 2013):

*“I can work 75%, and that is not a major issue in the sense that health is more important, but it has a major impact on my life”* (Boons et al., 2018, p647)

Conversely, living with CML was reported as having little impact on daily life by fewer patients, often after treatment had started (Chen et al., 2014; Guilhot et al., 2013; Lim et al., 2017). Patients also described how their disease and treatment affected family and friends and how they perceived the practical and psychological support from these groups as vital (Graffigna et al., 2017):

*“My family was badly affected by my disease. They were shocked at first, but as time went by they became such an important support for me.*” (Graffigna et al., 2017, p. 2747)

### 4.3 Changing perspectives

Only two publications referred to the ‘patient journey’ (Graffigna et al., 2017; Guilhot et al., 2013), although all noted changing perspectives according to time since diagnosis. The early post-diagnostic period was defined by ‘shock’, ‘anxious alert’ (described as a heightened awareness of their health) or ‘crisis’, with some patients saying they felt pessimistic and fearful (Graffigna et al., 2017; Guilhot et al., 2013):

*“I hyper-scrutinized my body in search of new symptoms or signals that my health was worsening. At that stage I was certain that ‘the worst’ was still to come.*” (Graffigna et al., 2017, p2749, )

This was followed by a process of adaptation, involving the dissipation of anxious feelings, before disease/treatment acceptance (Bolarinwa et al., 2018; Boons et al., 2018; Chen et al., 2014; Eliasson et al., 2011; Graffigna et al., 2017; Guilhot et al., 2013; Lim et al., 2017; Wu et al., 2015). Adaptation was an active process, involving growing knowledge and understanding of disease, increased awareness of blood results showing treatment response, and activity adjustments:

*‘‘It was all about the children before, educating and dressing them … Now I pay attention to myself more. I listen to myself and to what my body says.*” (Guilhot et al., 2013, p89)

One study reported patients finding it easier to talk about their disease once they had reached acceptance, with people diagnosed more recently saying they found adaptation easier, possibly due to the availability of effective treatments, with better prognosis (Guilhot et al., 2013). Some patients said they had gained more positive perspectives and felt ‘lucky’ they had CML rather than a more acute cancer (Bolarinwa et al., 2018; Graffigna et al., 2017; Guilhot et al., 2013; Wu et al., 2015):

*“There's a lot more people worse off than me so [I] don't complain too much.*” (Wu et al., 2015, p259)

Judging themselves as more fortunate was described as ‘downward comparison’ and was thought to lead to reluctance among some patients to seek help from HCPs (e.g. for side effects), (Wu et al., 2015). In contrast, patients also reported continuing feelings of fear and sadness:

*“I think I've adjusted to CML. Although to be honest I have to say that I still sometimes feel sad.*” (Graffigna et al., 2017, p2749)

Some patients developed a more negative perspective over time due to their experience of side effects:

*“In the course of time of treatment, patients developed more negative beliefs about TKI due to side effects (e.g. “nasty pills, “a drama*”) (Boons et al., 2018, p648, author quotation).

As patients achieved a ‘new normal’ (Guilhot et al., 2013) following acceptance and adaptation, they were said to renew life plans, such as marriage, friendships and hobbies (Graffigna et al., 2017; Guilhot et al., 2013). Patients expressed feelings which were optimistic, such as hoping to stop treatment in due time (Boons et al., 2018; Graffigna et al., 2017; Wu et al., 2015), but also feelings of fear for the future (Boons et al., 2018; Chen et al., 2014; Graffigna et al., 2017; Guilhot et al., 2013):

*“The idea of no longer responding is worrisome and you wonder about it when you have a chronic disease.’’* (Guilhot et al., 2013, p90)

## 5 Theme 2: disease management strategies

This theme captures patient behaviour (disease management and awareness, adherence, management of side effects), at an individual level and in the context of external influences, such as practitioner advice and drug availability.

### 5.1 Patients have their own management strategies

Patients described many strategies used to help them take their TKI medication, including routine/forward planning, often with family support (Bolarinwa et al., 2018; Boons et al., 2018; Eliasson et al., 2011; Guilhot et al., 2013; Lim et al., 2017; Wu et al., 2015):

*“My husband reminds me to take my drug; at times my phone ring[s] when it gets to the time to take it, I have never missed it …”* (Bolarinwa et al., 2018, p198)

Patients also developed various techniques to manage symptoms/side effects, such as taking medication around mealtimes or before going to bed, to reduce the effects of nausea (Chen et al., 2014; Eliasson et al., 2011; Lim et al., 2017; Wu et al., 2015):

*‘‘I changed to take the medicine before bed-time or after a meal. If I take it with an empty stomach, I will definitely vomit it out in ten minutes.*” (Chen et al., 2014, p124)

However, whilst data suggest some patients were willing to consult HCPs about disease related issues, such as stopping medication, the opposite appeared more common regarding side effects or adherence (Eliasson et al., 2011; Lim et al., 2017; Wu et al., 2015); meaning that HCPs could be unaware of difficulties. Reasons given by patients for non-consultation included reluctance to bother HCPs and/or patients considering their symptoms trivial. Similarly, patients were unlikely to inform HCPs about missed medication, thinking it was not important, not wanting to upset their doctor, or they could judge themselves whether a consultation was required (Eliasson et al., 2011; Lim et al., 2017; Wu et al., 2015):

*“I forgot to take the medicine with me. I'm a little bit worried, but I say no it's too late now and I don't want to tell the doctor, I don't want to upset the doctor*”. (Wu et al. p.258)

*“I was unable to hear for about a week, so I self-adjusted the dose …. I did not seek the consultation from doctors because my next clinic visit was 3 months after that.*” (Lim et al., 2017, p1927)

Some patients reported using complementary and alternative medicines to deal with side effects or for general health, such as herbal preparations and vitamin supplements (Bolarinwa et al., 2018; Wu et al., 2015). Such medicines were also sometimes used as an alternative to TKIs for those who held a strong belief in traditional medicine or when specialist care coverage was inadequate, causing a delay in diagnosis or interruption in TKI treatment (Bolarinwa et al., 2018; Tan et al., 2017):

*“I was very ill, I could not stand and I have no blood that my husband took me to several hospitals and herbalist homes with no relief.*” (Bolarinwa et al., 2018, p197)

### 5.2 Patients make their own decisions about adherence

Some patients occasionally decided to omit their TKI medication intentionally, often to avoid side effects. This enabled them, for example, to eat and drink normally on social/religious occasions or during periods of illness, which could be further complicated by medication that involved fasting prior to administration (Bolarinwa et al., 2018; Boons et al., 2018; Chen et al., 2014; Eliasson et al., 2011; Lim et al., 2017; Tan et al., 2017; Wu et al., 2015):

*“… I thought there was no way I was going [on holiday] and being tired. So I did actually stop taking the tablets for a week before I went …”* (Eliasson et al., 2011, p629)

Some reported feeling better after missing TKI medication, as side effects were absent (Eliasson et al., 2011). Unintentional non-adherence was also reported, commonly due to simple forgetfulness, often caused by a change in routine, travelling or social occasions (Bolarinwa et al., 2018; Boons et al., 2018; Eliasson et al., 2011; Graffigna et al., 2017; Lim et al., 2017; Tan et al., 2017; Wu et al., 2015):

*“My drug is my life, I try to follow the dosage on the doctor's prescription, but it might sometimes happen that I forget.*” (Graffigna et al., 2017, p2746)

Patients' beliefs about their medication affected motivation to adhere (Bolarinwa et al., 2018; Chen et al., 2014; Eliasson et al., 2011; Lim et al., 2017; Wu et al., 2015). Some reported fear of progression, others described themselves as ‘conformists’ who strictly followed medical advice, or said they had ‘faith’ in their doctor and treatment (Eliasson et al., 2011; Wu et al., 2015):

*“… It's a belief really, that's keeping me going. I've now put all my faith in [the imatinib]. From day one I've got faith in [my clinician].*” (Eliasson et al., 2011, p629)

Beliefs and misunderstandings about TKI medication could also result in non-adherence; for example, a fear of long-term effects or believing TKIs are only required if symptomatic (Chen et al., 2014; Tan et al., 2017):

*“I'm not sure about taking this medication, I feel well.*” (Tan et al., 2017, p1031)

Whilst some patients adhered because they did not experience side effects, others did so despite side effects (Eliasson et al., 2011). Data from one publication suggests adherence can change over time (Eliasson et al., 2011) being initially poor as individuals ‘got used to’ the medication, or decreasing over time, as motivation to adhere decreased, and response to medication had been achieved.

When faced with the decision of how to compensate for missed medication, some said they always took their treatment as soon as they remembered (usually the same day), whilst others reported not taking missed dose(s). Reasons patients did not compensate for missed doses included: thinking the missed dose would not affect response; feeling they could judge for themselves whether to change doses; not wanting to bother their doctor; or simply being unable to remember if they had taken a tablet or not (Boons et al., 2018; Eliasson et al., 2011; Lim et al., 2017; Tan et al., 2017; Wu et al., 2015)”

*“I get into the car, due to take off and remember about that, and I say, ‘Ah, only one day’; don't worry about that.*” (Wu et al., 2015, p258)

### 5.3 External influences on disease management

Decisions about adherence were made within the context of health and social systems. Unintentional non-adherence could also be due to prescription errors, difficulties with pharmacy (Eliasson et al., 2011) or problems accessing medication, and in certain countries (Nigeria and Malaysia), the costs of disease monitoring (Bolarinwa et al., 2018; Tan et al., 2017). Communication issues were cited as a barrier to TKI adherence, with some patients unable to gain access to advice (Eliasson et al., 2011; Wu et al., 2015):

*“…I guess because you don't want to get told off for not taking it, you know. And [if I take my imatinib or not] is not something I've been specifically asked either.”* (Eliasson et al., 2011, p629)

In some countries (e.g. Nigeria, Malaysia, Brazil and Russia), a limited supply of TKIs or out of pocket costs, such as laboratory costs and long journeys to hospital appointments, could affect adherence (Bolarinwa et al., 2018; Guilhot et al., 2013; Tan et al., 2017):

*“Before [this] my blood test BCR-ABL is free, now I need to pay hundred[s] over. For private [care], we struggle*” (Tan et al., 2017, p1032)

Although the synthesis indicated that high levels of adherence are encouraged by HCPs, there is also evidence that HCPs may unintentionally reinforce non-adherence by reassuring patients that “missing the odd dose” is acceptable (Bolarinwa et al., 2018; Eliasson et al., 2011; Wu et al., 2015):

*“I've missed a couple of nights and I've rang like the research nurse and she said, ‘Look, don't stress. It's only one night’*.” (Wu et al., 2015, p260)

Patients may also interpret ‘stable response’ to mean missing medication is safe (Bolarinwa et al., 2018; Boons et al., 2018; Eliasson et al., 2011):

*“Some patients perceived that the missed dose would have no effect on their TKI response and they argued that their haematologist also sometimes said to stop treatment for a period when experiencing side effects …”* (Boons et al., 2018, p648, author quotation)

The extent to which support was provided around adherence and the management of side effects differed between publications (Bolarinwa et al., 2018; Boons et al., 2018; Chen et al., 2014; Eliasson et al., 2011; Guilhot et al., 2013; Wu et al., 2015); and as already noted, conflicting advice could be given about missing medication (Eliasson et al., 2011; Wu et al., 2015):

*“Twelve out of 21 patients made comments in relation to receiving feedback that seemed to have reinforced the belief that ‘occasional’ non-adherence did not matter.*” (Eliasson et al., 2011, p628, author quotation)

Some data suggest that lack of awareness among HCPs about the extent of non-adherence could be due to their reliance on blood-monitoring rather than asking patients (Eliasson et al., 2011; Wu et al., 2015). Patients also said little advice was provided about if/how to compensate for missed medication and often made this decision themselves (Eliasson et al., 2011; Lim et al., 2017; Wu et al., 2015). Patients also indicated that advice on managing side effects could also be lacking (Boons et al., 2018; Wu et al., 2015):

*“When I vomited, the information wasn't there; do I take another dose, don't I, will I overdose?*” (Wu et al., 2015, p260)

### 5.4 Varying patient knowledge and information needs over time

Patient knowledge and understanding was said to influence disease management, including side effects, adherence and reporting to HCPs (Chen et al., 2014; Eliasson et al., 2011; Graffigna et al., 2017; Lim et al., 2017; Wu et al., 2015). Some patients showed awareness about CML. More, however, lacked knowledge, particularly about treatment (Boons et al., 2018; Chen et al., 2014; Eliasson et al., 2011; Lim et al., 2017; Wu et al., 2015). Misunderstandings included thinking medication was ‘stored’ in the body (Wu et al., 2015), being unclear on indicators of progression and not fully understanding monitoring:

*“… the nurse insisted that I need to have a regular check, that's strange, I can't see why it's necessary*.” (Chen et al., 2014, p123)

Some patients wanted HCPs to interpret their blood results (Guilhot et al., 2013; Wu et al., 2015), while others preferred to be involved themselves:

*‘‘I get the results personally, read them first, and bring them to my doctor.*” (Guilhot et al., 2013, p85)

Boons et al. (2018) reported that patients expressed a need for information to be current and presented in an honest, understandable format, including written material. There was a particular need for more information on side effects, including impact on sexuality. Patients also wanted more information about hospital appointment systems and social support:

*“It should be honest, I want to know exactly what to expect*” (Boons et al., 2018, p647)

Guilhot et al. (2013) described patient need for information at each stage in the ‘CML journey’, saying only basic disease/treatment understanding was needed during the initial ‘crisis’/’shock’ phase; with more detail required during ‘adaptation’. Disappointment amongst patients was noted, concerning how little information clinicians offered at this time:

*“Patients said that their HCPs provided little to no guidance on how to properly take their therapy and that they implemented their own methods to standardize their drug-taking routines.*” (Guilhot et al., 2013, p88, author quotation)

Upon reaching the ‘new normal’, patients' anxieties reduced and the need for information was said to be minimal (Guilhot et al., 2013).

## 6 Theme 3: valued aspects of care

This theme describes factors valued by people with CML about their care, and potential improvements suggested by patients and HCPs.

### 6.1 Factors valued by patients and HCPs

Importantly, rather than education, patients appeared to place greater value on psychological support, offered by HCPs who were accessible, had a caring attitude and would provide reassurance (Bolarinwa et al., 2018; Boons et al., 2018; Eliasson et al., 2011; Guilhot et al., 2013; Lim et al., 2017). The importance of trust and ‘faith’ in HCPs was also discussed (Eliasson et al., 2011; Guilhot et al., 2013; Lim et al., 2017):

*“I was shocked when I was first diagnosed with this disease, but my doctor gave me encouragement. He assured me that this medication will help me, so I felt more relaxed.*” (Lim et al., 2017, p1927)

*“my doctor make[s] sure I get it even during doctor's strike, he also calls me to find out how I am doing.*” (Bolarinwa et al., 2018, p197)

*‘‘I feel that I am in very good hands. I trust my doctor fully.’’* (Guilhot et al., 2013, p85)

Interestingly, more recently diagnosed CML was described by some patients and their HCPs as ‘low key’, in that it was a chronic disease, treatable with low-intensity oral medication. (Chen et al., 2014; Guilhot et al., 2013 Wu et al., 2015):

*“Another patient was “happy knowing there's a pill [she] can pop” (PT7), noting that other potential treatments were associated with reduced efficacy or greater toxicity.” (*Wu et al., 2015, p259)

*“The first doctor … said that it was leukemia but I should not be worried because medicine is very developed nowadays,’’* (Guilhot et al., 2013, p88, p88)

Whilst this depiction of CML could alleviate anxiety for some, it could also suggest to patients that they should be able to manage their CML themselves, thus contributing to disinclination to consult HCPs.

*“ I can judge it by myself, as I know my condition very well. If I have a flu or fever, I will reduce the dose by myself.*” (Lim et al., 2017, p1927)

### 6.2 Interpersonal and resource-based improvements in care

Several papers suggested patient/HCP consultations could be more open and individualised (Eliasson et al., 2011; Graffigna et al., 2017; Wu et al., 2015), with better advice on TKI treatment options (Chen et al., 2014; Guilhot et al., 2013), managing side effects (Boons et al., 2018; Guilhot et al., 2013; Lim et al., 2017), dealing with omitted doses (Chen et al., 2014; Eliasson et al., 2011; Wu et al., 2015), monitoring response (Guilhot et al., 2013) and establishing drug-taking routines (Eliasson et al., 2011; Guilhot et al., 2013; Tan et al., 2017) Supportive, non-judgemental and open dialogue, taking account of the patient's personal ‘narrative’, was also recommended to encourage the sharing of anxieties and adherence behaviour. This reflected patients' accounts of what they value in their HCP:

*“… open communication will be beneficial to the patient in the management of CML throughout his or her journey.*” (Guilhot et al., 2013, p91, author quotation)

Regarding resources, data indicated that input was lacking from community services, with patients saying their General Practitioner (GP) and local pharmacists had little knowledge of CML (Eliasson et al., Wu et al., 2015). Suggested improvements included more clinic staff and training people with CML as ‘counsellors’ for other patients, (Bolarinwa et al., 2018). With respect to facilities and costs, longer-term prescriptions were suggested by both patients and HCPs (Chen et al., 2014):

*“a two-week schedule just passes too quickly, we should be allowed to have a long-term drug supply and only come to visit the doctor when we don't feel right.’’* (Chen et al., 2014, p124)

## 7 Discussion

The nine qualitative studies included in this thematic synthesis clearly show that CML can have a significant impact on physical and psychological well-being and daily activities. TKI treatment side effects, traditionally physician assessed and reported as mild to moderate in clinical trials (Baccarani et al., 2014; Efficace and Cannella, 2016; Flynn and Atallah, 2016), were found to be widespread and disruptive. Interestingly, within work to develop and test CML specific patient reported outcomes measures, other authors report that the majority of patients with CML experienced persistent symptoms, ranging from mild to severe (Williams et al., 2013; Zulbaran-Rojas et al., 2018). It has been suggested that such long-term symptom burden may be more difficult to tolerate than intensive treatment, given short-term with curative intent (Frick et al., 2017). As previously noted, living with CML is also related to significantly worse health related quality of life (HRQOL) (Efficace et al., 2013; Williams et al., 2013; Zulbaran-Rojas et al., 2018), than found in the general population (Efficace et al., 2011; Phillips et al., 2013). In response, validated CML specific HRQOL and symptom burden questionnaires have been developed (Efficace et al., 2014; Williams et al., 2013), signifying a move away from physician assessed side-effects to patient reported outcome measures.

Our synthesis highlights the chronicity of CML and evidences patients gradually developing strategies, beliefs and decision-making processes to manage their disease, adherence and side effects; often without consultation with hospital clinicians and sometimes without a thorough understanding of their treatment. This is potentially relevant to other cancers managed with oral medication, which represents around 25% of all current cancer treatments in the United States of America (USA) (Abbott et al., 2014; Weingart et al., 2008). This shift from hospital based intravenous therapy to self-managed home treatment has many similarities with chronic illnesses, such as diabetes and cardiovascular diseases, which also tend to be self-managed.

Aspects of self-management in chronic illness, such as adherence to medication are widely documented (Velde et al., 2019). The multifactorial nature of non-adherence to medication in chronic disease as a global burden has been well described in a key WHO report (Sabaté, 2003) and consequent literature. Less well documented are definitions of chronic cancer and patient experiences of chronic cancer, including their disease management and hospital care (Harley et al., 2019; Pizzoli et al., 2019). Interestingly, patient reluctance to seek clinician advice regarding non-adherence and side effects identified in the current study, is corroborated in one of few studies on chronic cancer experience (Harley et al., 2019), and a large survey highlighting unmet needs among CML patients (Breccia et al., 2015).

Our study provides insight and understanding into the complexities CML patients face, contributing context to what is already known. It highlights how patients may lack knowledge about treatment; change their perspective on life; and the influence of HCPs in terms of the way they deliver care and advice. Regarding healthcare systems, it describes the possibility of hospital errors, pharmacy delay and blood monitoring issues. Other authors suggest further complexity due to adherence being underpinned by several factors, including side effects, co-morbidities and physician characteristics (Darkow et al., 2007; Marin et al., 2010; Noens et al., 2009). The multifactorial nature of chronic cancer symptoms is also said to contribute (Frick et al., 2017; Zulbaran-Rojas et al., 2018) with fatigue, for example, not only relating to treatment, but also psychological distress, physical side effects (e.g. pain), and the impact of these on daily life (e.g. ability to work) (Efficace et al., 2013; Hofman et al., 2007; Zulbaran-Rojas et al., 2018).

Additional complexity is introduced by HCPs if they inadvertently provide conflicting or misguided advice to patients; are unaware how individuals cope with treatment and side effects; or do not provide sufficient or consistent psychological support. Wu et al. (2015) highlight complexity of care delivery from the perspective of HCPs, with issues such as budget and time restraints preventing adequate support, and language issues and miscommunication between hospital departments effecting adherence. This study also reports alignment between HCPs and patients regarding the late identification of side effects and perceptions of CML as a low maintenance disease. In recognition of such complicated pathways and experiences, and the impact of health system factors, Harley et al. (2019) developed the Chronic Cancer Experience Questionnaire (CCEQ), which includes multidimensional questions on side effects and daily activities, but also psychological wellbeing and the use of clinical services and available support.

Given the complexity of CML, its increasing prevalence in the TKI era, and emerging evidence of unmet needs, it is important that adequate care and support is available during long-term survivorship. Although this phase is well documented (Department of Health, 2013; Mayer et al., 2014; McCabe et al., 2013), much available literature refers to the time-period ‘beyond’ treatment, with little addressing experiences of ‘living with’ chronic cancers whilst taking continual oral medication, as occurs with CML. This concurs with results from a study in the USA (Frick et al., 2017), where fewer survivorship care plans were reported for patients with chronic cancer (including CML), than for those treated with curative intent.

Although a review of haematology survivorship models identified a diverse range of programmes and suggested primary care HCP involvement (rather than haematology alone or another single discipline), the models were said to lack measures of effectiveness (Taylor et al., 2015). Unclear professional responsibilities, lack of skills and educational resources, and (concurrent with this synthesis), insufficient time, have all been identified as barriers to nurses providing adequate care during survivorship for patients with haematological malignancies (Langbecker et al., 2016; Wallace et al., 2015). Unfortunately, this is associated with a lack of studies addressing self-management interventions for cancer patients in general (Howell et al., 2019), despite considerable literature focusing on factors effecting self-management and the impact of such interventions in chronic disease (McBain et al., 2015; Schulman-Green et al., 2016; Vassilev et al., 2011).

This is the only qualitative synthesis to generate evidence about experiences of living with CML and adhering to prescribed medication. Consequently, we are unable to compare our findings with similar work. Major strengths include a robust search strategy, last updated in September 2019; inclusion of 320 patients; two researchers checking study eligibility, codes and themes; and use of NVIVO computer software to facilitate data management and retrieval. The studies we included originated from different countries, some of which described systems of free access to TKI medication, and others that did not clarify this. However, as all the studies had inclusion criteria that the patient was receiving a TKI medication, presumably those patients in the studies all had access to their medication. Also, as findings were relatively consistent across studies, we expect our analysis is largely transferable to other regions, where patients access TKIs. The synthesis may be limited by the exclusion of grey literature and articles not written in the English language, which could not be fully searched due to time-constraints; however, the authors were not aware of any ongoing work that might impact on study findings.

Included studies (Table 3) also had limitations. Overall, several lacked a thorough reporting of methods, particularly sampling strategies (e.g. inclusion criteria and reporting on excluded participants), and in the application of theoretical models to data analysis. For example, Wu et al. (2015) used interpretative phenomenological analysis (IPA), but did not describe how its features were implemented in the analysis, including the impact of the researchers' own conceptions on the findings. Strengths were mostly in the reporting of results. Although this varied from descriptive to more conceptual accounts, there was consistency between the data and results, quotations were used appropriately, and findings were generally presented clearly.

Regarding clinical implications, unmet need and outcomes can be appropriately measured using the CCEQ. Survivorship programmes, individualised and developed for patients with CML, would provide the opportunity for discussions about side effects and adherence, enabling HCPs to understand the patient's perspective and understanding, and meet educational requirements, as necessary. Such care should be supported by systems that allow adequate time and resources for this, with a defined role for primary care HCPs, including GPs and practice nurses. Crucially, of greatest value to patients is a caring approach among HCPs, supported by the creation and maintenance of a culture of kindness and compassion (Campling, 2015).

Development of survivorship programmes or other interventions to support self-management in CML requires further qualitative research to investigate the experiences of those caring for people with CML. This should also examine contextual issues for patients, such as social support, views on hospital care and disease knowledge. Recent publications have begun to emerge that suggest some patients may now safely discontinue TKI medication (Clark et al., 2017; Etienne et al., 2017; Saussele et al., 2018). Further qualitative research exploring the experiences of such patients, alongside the QOL measures used in these studies, will add depth to our understanding of this new challenge for patients.

## 8 Conclusion

This synthesis has demonstrated the significant impact CML and TKI treatment have on patient wellbeing and day to day life. As with an increasing number of cancers, CML involves the self-management of treatment at home, outside the clinical environment. Our synthesis provides evidence that, in the home-setting, patients develop their own strategies to manage adherence and side effects, often not discussing this with HCPs. CML self-management occurs within the context of the individual's own knowledge and perceptions of their disease, as well as the influence of their HCP and the nuances of the health system providing care. As in other chronic cancers, little research exists about experiences and survivorship in CML, or the perspectives of HCPs. However, given that treatment is administered at home, the development of survivorship programmes or interventions should perhaps look beyond a medical model of disease management, to a more a community-based social model, delivered with the support of primary care teams, in a setting familiar to patients and where they live their lives. Such an approach, which has the capacity to adapt to individual contexts and choices, may be most appropriate to develop mechanisms for supporting patient decision making and disease management strategies.

## Disclaimer

This paper presents independent research supported by Bloodwise (Grant No. 15037). The views expressed are those of the authors and not necessarily those of the funder.

## Declaration of competing interest

All the authors declare they have no conflict of interest that could inappropriately influence this study.
